# Supplementary material for: Content-rich biological network constructed by mining PubMed abstracts
Source: BMC Bioinformatics. 2004 Oct 8;5:147. doi: 10.1186/1471-2105-5-147 (PMC528731; doi:10.1186/1471-2105-5-147)
Supplement: Additional File 5 — The original Chilibot query results of the term "long-term potentiation (LTP)" and 22 other terms, limiting the latest references analyzed to the years 1990, 1995, 2000, and 2004. [file 1471-2105-5-147-S5.bz2 › chilibotAdditionalFile5/ltp2004/html/AMPA_CAMKII.html]

 


 **AMPA** and **CAMKII** 
  
Found 64 abstracts in PubMed,  **30 abstracts were retrieved and analyzed**.  


---

 Search Google  |
 PDF files only 
|  EDU domain only 

---

**Interactive relationship** (e.g. stimulation, inhibition, etc)

- Activation of  **CaMKII**  has been shown to phosphorylate the glutamate receptor 1 subunit of the  **AMPA**  receptor AMPAR, thereby affecting some of the properties of the receptor.  Ref: 12040047 J Neurosci, 2002
- Furthermore, the serotonergic modulation of  **AMPA**  currents was occluded by application of the Ca calmodulin dependent kinase II inhibitors and blocked by intracellular injection of calmodulin or recombinant  **CaMKII** .  Ref: 12149253 J Biol Chem, 2002
- In the simplest model for long term potentiation,  **CaMKII**  is activated by calcium influx through NMDA N methyl D aspartate receptors and then potentiates synaptic efficacy by inducing synaptic insertion and increased single channel conductance of  **AMPA**  alpha amino 3 hydroxy 5 methyl 4 isoxazole propionic acid receptors.  Ref: 11459059 Nature, 2001
- We show that Ras relays the NMDA R and  **CaMKII**  signaling that drives synaptic delivery of  **AMPA**  Rs during long term potentiation.  Ref: 12202034 Cell, 2002
- A model of synaptic memory a  **CaMKII**  PP1 switch that potentiates transmission by organizing an  **AMPA**  receptor anchoring assembly.  Ref: 11502252 Neuron, 2001
- Recent cell biological, genetic, and physiological analyses suggest that one of the cellular explanations for LTP and  **CaMKII**  function might be the trafficking of  **AMPA**  type receptors to synapses in response to neural activity.  Ref: 11891759 Bioessays, 2002
- Synaptic accumulation of  **AMPA**  receptors containing the glutamate receptor 1 subunit can be driven by calcium calmodulin dependent protein kinase II  [ **CAMKII** ]  activity or long term potentiation and requires an interaction between GluR1 and a type I PDZ domain containing protein.  Ref: 12097490 J Neurosci, 2002
- Recent studies have shown that phosphorylation of  **AMPA**  receptors by cAMP dependent protein kinase PKA and calcium and calmodulin dependent protein kinase II  **CaMKII**  potentiates their activity, but phosphorylation of the receptor subunits may also affect their interaction with intracellular proteins, and their expression at the plasma membrane.  Ref: 11059799 Neurochem Res, 2000
- The ligand induced increases were i blocked by inhibitors of Src tyrosine kinase, antagonists of N methyl d aspartate receptors, and inhibitors of calcium calmodulin dependent protein kinase II  [ **CAMKII** ]  and ii accompanied by phosphorylation of both the Thr 286 site on calmodulin dependent protein kinase II and the Ser 831 site on the GluR1 subunit of the  **AMPA**  receptor.  Ref: 12524441 J Biol Chem, 2003
- The regulation of  **CaMKII**  levels uses distinguishable pathways, one responsive to NMDA receptor blockade that controls alphaCaMKII alone, the other responsive to  **AMPA**  receptor blockade and involving betaCaMKII and possibly further downstream effects of betaCaMKII on alphaCaMKII.  Ref: 12495625 Neuron, 2002

**Parallel relationship** (e.g. studied together, co-existance, homology, etc.)

- As a direct consequence of alpha  **CaMKII**  association, we found increased GluR1 delivery to cell surface in cultured hippocampal neurons paralleled by  **AMPA**  dependent increase in Na I levels.  Ref: 12372013 Eur J Neurosci, 2002
- The results of the present study suggest that 2 pyrrolidinone persistently enhances activity of the calcium permeable  **AMPA**  receptors, GluR and GluR, 3, by interacting with  **CaMKII** .  Ref: 11834304 Brain Res Mol Brain Res, 2002
- The enhanced learning correlates with increased phosphorylation of cyclic AMP dependent response element binding CREB protein, of calcium calmodulin dependent protein kinase II  **CaMKII**  and of the GluR1 subunit of the  **AMPA**  receptor.  Ref: 12198546 Nature, 2002
- In addition, both  **CaMKII**  specific inhibitor KN 93 and  **AMPA**  receptor antagonists CNQX and NBQX rescued neuronal survival to control values.  Ref: 12372013 Eur J Neurosci, 2002
- Application of serotonin or 5 HT agonists to PFC slices reduced  **CaMKII**  activity and the phosphorylation of  **AMPA**  receptor subunit GluR1 at the  **CaMKII**  site in a PP1 dependent manner.  Ref: 12149253 J Biol Chem, 2002
- Within the framework of a model involving calcium calmodulin dependent protein kinase II  [ **CAMKII** ]   **CaMKII** , calcineurin PP2B and type 1 protein phosphatase PP1, we aimed at delineating the conditions allowing a biphasic U shaped relationship between  **AMPA**  R and calcium signal amplitude, and thus bidirectional plasticity.  Ref: 12823459 Eur J Neurosci< MedlineTA>Eur J Neurosci, 2002
- PKC activation induced a simultaneous translocation of calcium calmodulin dependent kinase II  **CaMKII**  to synapses but no change in spine number, presynaptic terminal number, or the distribution of  **AMPA**  receptors or the synaptic scaffolding protein PSD 95.  Ref: 11896155 J Neurosci, 2002
- Because  **AMPA**  and NMDA receptors, as well as l type calcium channels are calcium permeable, we also investigated the role of the calcium activated second messenger calcium calmodulin dependent protein kinase II  [ **CAMKII** ]  CaM KII.  Ref: 14725635 Eur J NeurosciEur J Neurosci, 2002
